# Relative Expression Levels Rather Than Specific Activity Plays the Major Role in Determining *In Vivo* AKT Isoform Substrate Specificity

**DOI:** 10.4061/2011/720985

**Published:** 2011-08-22

**Authors:** Rachel S. Lee, Colin M. House, Briony E. Cristiano, Ross D. Hannan, Richard B. Pearson, Katherine M. Hannan

**Affiliations:** ^1^Growth Control and Differentiation Program, Trescowthick Research Laboratories, Peter MacCallum Cancer Centre, Melbourne, VIC 8006, Australia; ^2^Cancer Genomics Genetics Laboratory, Peter MacCallum Cancer Centre, East Melbourne, VIC 8006, Australia; ^3^Cephalon Australia Pty Ltd., Parkville, VIC 3052, Australia; ^4^Department of Biochemistry and Molecular Biology, University of Melbourne, Melbourne, VIC 3010, Australia; ^5^Department of Biochemistry and Molecular Biology, Monash University, Clayton, VIC 3168, Australia

## Abstract

The AKT protooncogene mediates many cellular processes involved in normal development and disease states such as cancer. The three structurally similar isoforms: AKT1, AKT2, and AKT3 exhibit both functional redundancy and isoform-specific functions; however the basis for their differential signalling remains unclear. Here we show that *in vitro*, purified AKT3 is *∼*47-fold more active than AKT1 at phosphorylating peptide and protein substrates. Despite these marked variations in specific activity between the individual isoforms, a comprehensive analysis of phosphorylation of validated AKT substrates indicated only subtle differences in signalling via individual isoforms *in vivo*. Therefore, we hypothesise, at least in this model system, that relative tissue/cellular abundance, rather than specific activity, plays the dominant role in determining AKT substrate specificity *in situ*.

## 1. Introduction

The AKT protooncogene comprises a family of three highly homologous serine/threonine kinases (AKT1, 2 and 3) [1], that play major regulatory roles in a wide range of cellular processes including cell survival, growth, proliferation, angiogenesis, and metabolism [2–4]. These processes are key to normal development and often dysregulated in disease. Consistent with these wide-ranging roles, to date, more than 100 AKT substrates have been identified that are integral in the regulation of one or more of these cellular processes [3]. 

Each AKT isoform consists of a pleckstrin homology domain (>75% identity), a linker region (>17% identity), a kinase domain (>87% identity), and a carboxyl tail with a hydrophobic motif (>66% identity) [1]. The two key regulatory phosphorylation sites on AKT are also conserved, Ser473 and Thr308 in AKT1, Ser474 and Thr308 in AKT2, and Ser472 and Thr305 in AKT3 [5]. Thr308 is phosphorylated by 3-Phosphoinositide-dependent kinase 1 (PDK1) and Ser473 by mammalian target of rapamycin (mTOR) complex 2 (mTORC2) [6–9]. Recent studies have suggested that phosphorylation at Ser473 dictates AKT signaling to a specific set of substrates, and phosphorylation at Thr308 determines the kinase activity [8, 10].

Whilst all three AKT isoforms share a high sequence identity and exhibit functional redundancy, there is also genetic evidence that they can function distinctly. This is illustrated in the distinct phenotypes exhibited by the single isoform knockout (KO) mice and the severe phenotypes exhibited by double KO (dKO) mice. AKT1^−/−^ mice are not 100% viable, with surviving mice that reach adulthood being 15–20% smaller than their wild type (wt) and heterozygous counterparts [11]. Furthermore, AKT1^−/−^ mice exhibit a smaller brain and liver as a result of a reduction in cell number when compared to wt mice [12] indicating that AKT1 is important in regulating proliferation. Heart size was also reduced; however it was caused by a decrease in cell size [12], suggesting that AKT1 predominantly affects cell growth in this tissue. The AKT2^−/−^ mice exhibited a very different phenotype, reminiscent of mice with Type 2 diabetes mellitus, suggesting a specific role for this isoform in glucose metabolism [13] although more recent studies in AKT1^−/−^ mice have indicated that both AKT1 and 2 are required for the regulation of glucose homeostasis in the periphery [14]. AKT3^−/−^ mice are not growth retarded but show a 25% reduction in brain size due to decreased cell number and a smaller cell size [12]. The phenotypes of AKT dKO mice were more severe than that observed in single KO mice. AKT1/2^−/−^ mice die shortly after birth and suffer from severe dwarfism, impaired skin development, skeletal muscle atrophy, impaired adipogenesis, and bone development [15], none of which was observed for the individual KO mice. The additive effect of AKT1 and AKT2 suggests a functional redundancy between these two isoforms. AKT1/3^−/−^ mice die between E11-E12, indicating the importance of AKT1 and AKT3 in postnatal survival [16]. AKT2/3^−/−^ mice are viable but smaller than their wt counterparts [17]. This less severe phenotype than the other compound mutants indicates a dominant role for AKT1, at least in development. 

Recent studies suggest that the different AKT isoforms can signal via distinct subsets of downstream pathways and that these subsets can vary depending on cellular and tissue context [12], reinforcing the need to investigate isoform-specific signaling in different cell types. For example, AKT3 but not AKT1 is crucial for activation of the mTORC1/S6K1 signaling pathway in brain [12]. Furthermore, cellular context has been seen to be important for AKT1 signaling where AKT1^−/−^ livers were smaller due to reduced cell number while AKT1^−/−^ hearts were smaller entirely because of reduced cell size [12]. In addition, evidence for distinct signaling pathways to and from individual AKT isoforms is beginning to accumulate. For example, reduction of PTEN expression in melanocytes activates AKT3 apparently without affecting the activity of AKT1 or 2 [18]. While the mechanism for this selective activation remains to be elucidated it provides an intriguing precedent.

Defining the mechanism(s) of differential signaling via these highly homologous AKT isoforms will be critical for understanding the role played by AKT in many essential cellular functions. It is clear that differential expression patterns can explain some of these functional roles. AKT1 is widely expressed in tissues including the brain, heart, lung, skeletal muscle, thymus, and skin while AKT2 is predominantly expressed in brown fat and the heart, and AKT3 was most abundantly expressed in the brain [16]. Moreover, AKT isoforms have been shown to localize to different subcellular compartments in a cell-line-specific manner [19, 20]. In unstimulated cells, AKT1 localised in the cytoplasm, AKT2 at the mitochondria, and AKT3 in the nucleus of breast, prostate, and liver cancer cell lines [20]. However, insulin-stimulated adipocytes demonstrated a higher level of AKT2, than AKT1, localization at the plasma membrane, thus linking AKT2 to the isoform-specific regulation of GLUT4 translocation and glucose metabolism [19]. 

In addition to variation in expression and localization patterns, differences in the intrinsic activity of the isoforms in phosphorylating synthetic peptide substrates have been reported [21–24]. Indeed, we have shown previously that purified AKT isoforms vary with their specific activity in phosphorylating model peptide substrates, with AKT3 being ~15-fold more active than AKT1 which was ~10-fold more active than AKT2 [25]. These findings were consistent with the findings of Walker et al. [24] who purified each isoform from HEK293 cells and further activated them by incubation with PDK1. In these experiments, a truncated form of AKT3 was 3-fold more active than AKT1 that was 5-fold more active than AKT2 [24]. Here we have examined whether these fundamental differences in enzyme kinetics contribute to substrate specificity *in vivo*.

Given the cell-type-specific regulation of isoform-dependent signaling described above, we chose to directly compare the intrinsic activity of individual AKT isoforms purified from HEK293 cells with their ability to phosphorylate *in vivo* substrates in the same cell type. HEK293 were chosen as they enable high levels of expression of exogenous AKT to allow purification of sufficient kinase to enable detailed kinetic analysis of enzyme activity. We focused our study on AKT 1 and 3 predominantly given the low activity of AKT2 after confirming this using isoforms immunopurified from HEK293 cells stimulated with serum, insulin, and pervanadate. We first confirmed that AKT3 was indeed more active than AKT1 in peptide kinase assays and against a newly identified protein substrate, ribosomal protein S7 (rpS7) (see supplementary Figure  1 in Supplementary Material available online at dois 10.4061/2011/720985; [26]). We have used gain- and loss-of-function approaches employing constitutively active forms of the isoforms and specific siRNAs to examine *in vivo* AKT isoform-specific signaling towards established direct AKT substrates involved in cell proliferation, survival, and growth including GSK3*α*/*β*, FoxO1/3a, MDM2, and PRAS40, and via the activation of the mTORC1 pathway leading to cell growth. In contrast to the marked differences in intrinsic enzyme activity between the AKT isoforms, only subtle differences in *in vivo* substrate specificity were observed. This thus suggests that AKT isoform-specific regulation of cellular function is, in a large part, dictated by the relative expression levels in the relevant cell type.

## 2. Material and Methods

### 2.1. Cell Culture and Treatments

Human embryonic kidney (HEK293) cells were cultured in Dulbecco's Modified Essential Medium (DMEM) plus 10%  (v/v) fetal bovine serum (FBS) and 1%  (v/v) antimycotic/antibiotic (Am/Ab, Invitrogen) and maintained at 37°C in 5% CO_2_. Cells were serum-starved (DMEM no FBS) for 24 hours then stimulated with 1  *μ*M insulin, 0.1 *μ*M pervanadate or 10% FBS for 20 minutes or pretreated with 5 *μ*M AKT inhibitor (AKTi, Calbiochem, # 124017), 20 nM rapamycin (Calbiochem, #553210), or both for 30 minutes then stimulated with 10% FBS for 20 minutes. 

### 2.2. Harvesting Cells for Protein Lysates

Proteins were harvested as described in [25]. Briefly, cells were lysed in Rac Lysis Buffer (RLB: 50 mM Tris-hydrochloride (Tris-HCl) pH7.5, 1% (v/v) NP-40, 120 mM sodium chloride (NaCl), 1 mM ethylenediaminetetraacetic acid (EDTA), 50 mM sodium fluoride, 40 mM *β*-glycerophosphate, 0.1 mM sodium vanadate, 1 mM benzamidine, complete EDTA-free protease inhibitor cocktail (Roche), and phosSToP phosphatase inhibitor cocktail (Roche)) and cleared by centrifuging (16000 ×g, 15 minutes, 4°C). Samples were snap frozen and stored at −80°C. The protein concentration was determined using the DC protein assay kit (BioRad). 

### 2.3. Expression and Purification of Recombinant AKT Isoforms

HEK293 cells were transfected via the calcium phosphate method [27] with pCDNA3 HA-tagged wtAKT1, wtAKT2, or pCMV5 HA-tagged wtAKT3 (Pearson Laboratory), pCDNA3 GST-wtAKT1 [28], pCMV5 GST-wtAKT3 [25], or pCDNA3 constitutively active myristoylated (myr) HA-tagged AKT1, AKT2, or AKT3 (Pearson Laboratory). Cells were treated and harvested into RLB and lysates cleared by centrifugation (14500 ×g, 15 minutes, 4°C). HA-tagged AKT isoforms were immunoprecipitated from cleared lysates (100 *μ*g) using Protein A Sepharose 4B beads (Zymed). GST-tagged AKT isoforms were purified by affinity chromatography as described in [28, 29]. Eluates were combined, buffer exchanged by overnight dialysis against 0.27 M sucrose in RLB, and purified GST-tagged wtAKT concentrated using Centricon 50s (Millipore), snapped frozen, and stored at −80°C. Samples were resolved by SDS-PAGE alongside known concentrations of bovine serum albumin (BSA), Coomassie R-250 stained, and the concentration determined by densitometry using Scion Image software (Scion Incorporation). 

### 2.4. Expression and Purification of HIS-rpS7

Human ribosomal protein S7 (rpS7, gift from Dirk Görlich, Heidelberg [30]) was cloned into the pQE-60 vector using restriction sites NcoI and BamHI and transformed into XL-Blue E.coli cells. Expression of HIS-rpS7 was induced with 1 mM isopropyl-*β*-D-thio-galactoside (IPTG) for 5 hours, cells pelleted (6000 ×g, 4°C, 10 minutes), resuspended in buffer A (300 mM NaCl, 50 mM sodium phosphate buffer, pH 7.0), and sonicated. The insoluble fraction was pelleted as above, resuspended in 20 mL of buffer B (8 M urea in buffer A), and sonicated. The clarified sample was tumbled with talon resin (Clontech) for 2 hours at room temperature (RT), washed 3 times with buffer B, and HIS-rpS7 eluted with 150 mM imidazole. HIS-rpS7 was dialysed sequentially for 4 hours at 4°C against: 4 M urea in PBS^−/−^; 2 M urea in PBS^−/−^, 20% glycerol and 0.5 mM phenylmethylsulphonyl fluoride (PMSF); 0.5 M urea in PBS^−/−^, 20% glycerol and 0.5 M PMSF; and twice in PBS^−/−^, 20% glycerol and 0.5 mM PMSF. Purified HIS-rpS7 was clarified by centrifugation (20000 ×g, 2 minutes, 4°C) and stored at −80°C. 

### 2.5. SDS-PAGE and Immunoblotting

Protein lysates (20–50 *μ*g) were resolved by sodium dodecyl sulphate polyacrylamide gel electrophoresis (SDS-PAGE) alongside Benchmark Prestained Protein Ladder (Invitrogen) or PageRuler Prestained Protein Ladder Plus (Fermentas). Proteins were then transferred onto immobilon-P membrane (Millipore) and immunoblotted with primary antibodies listed in Supplementary Table  1. Antimouse and antirabbit horseradish peroxidase-conjugated secondary antibodies (BioRad) were used at 1:2000 dilution. Signals were detected by enhanced chemiluminescence (Perkin Elmer) onto film and quantified by densitometry using ImageJ 1.42q (National Institutes of Health, USA). Paired *t*-test statistical analysis was performed using GraphPad Prism version 5.00, GraphPad Software, San Diego, Calif, USA, http://www.graphpad.com/.

### 2.6. Coomassie R-250 Staining

After SDS-PAGE, gels were stained with Coomassie R-250 (0.2% Coomassie R-250, 50% ethanol, 10% acetic acid) for 1 hour then destained with 25% ethanol plus 7% acetic acid. 

### 2.7. Knockdown of Endogenous AKT Isoforms

Individual AKT isoforms were silenced using On-Target plus SMARTpool small interfering RNAs (siRNAs) purchased from Dharmacon (AKT1: L-003000-00, AKT2: L-003001-00, AKT3: L-003002-00). Control siRNAs targeting enhanced green fluorescent protein (EGFP) were purchased from Sigma-Proligo (siEGFP 5′ sequence: 5′-GCAGCACGACUUCUUCAAGTT-3′, siEGFP 3′ sequence: 5′-CUUGAAGAAGUCGUGCUGCTT-3′). HEK293 cells at 50–60% confluency were transfected with 25 nM of siAKT1, siAKT2, or siAKT3 either individually or simultaneously using lipofectamine reagent (Invitrogen) following the manufacturer's instructions. After 4 hours, the media was replaced with DMEM plus 10% FBS and 1% Am/Ab then incubated overnight.

### 2.8. Direct Kinase Assay

Direct kinase assays were performed as described in [31]. Briefly, purified or immunoprecipitated AKT were incubated with [*γ*-^32^P]ATP and varying concentrations of RPRAATF peptide or HIS-rpS7 at 30°C for 20 minutes. The assay was terminated by spotting onto p81 paper, washed, dried, and [*γ*-^32^P] counted using the Tri-Carb 2100TR liquid scintillation analyzer (Skudtek Scientific, Pty Ltd). For whole cell lysates, the reaction was terminated by adding 10 *μ*L of 10% trichloroacetic acid. The sample was then cleared, spotted onto p81 paper, and counted as above. In the case of HIS-rpS7, samples were resolved by SDS-PAGE, the gel stained with Coomassie R-250, dried, the appropriate bands excised and counted as above. 

### 2.9. Two Dimensional Gel Electrophoresis (2DGE)

All chemicals were purchased from GE Healthcare or Sigma. Protein samples lysed in RLB were precipitated using the Ettan 2D Clean up Kit (GE Healthcare), resuspended in labelling buffer (7 M urea, 2 M thiourea, 4% CHAPS, 30 mM Tris) and the protein concentration determined using the Ettan 2D Quant Kit (GE Healthcare). Protein samples (50 *μ*g) were adjusted to pH 8.5 with 100 mM sodium hydroxide, labelled in the dark with 400 pmol of CyDye Fluor minimal dye Cy2 (GE Healthcare) for 30 minutes on ice and then quenched with 10 mM lysine. Labelled samples were combined with unlabelled proteins to a total of 250 *μ*g, adjusted to a final volume of 340 *μ*L (7 M urea, 2 M thiourea, 4% (w/v) CHAPS, 0.004% (w/v) bromophenol blue (BPB), 1% (w/v) DTT, and 1% (v/v) IPG buffer) and passively rehydrated onto an 18 cm, nonlinear pH 3–11 Immobiline DryStrip gel (GE Healthcare). Proteins were focused using the Ettan IPGphor3 (GE Healthcare) at 20°C with 50 *μ*A per strip using the following protocol: step and hold at 150 V for 3 hours, step and hold at 300 V for 3 hours, gradient to 1000 V for 6 hours, gradient to 10000 V for 1 hour, step and hold at 10000 V for 3 hours. Immobiline DryStrip gels were incubated in equilibration buffer (6 M Urea, 2% (w/v) SDS, 50 mM Tris·HCl pH 8.8, 0.002% BPB, 30% (v/v) glycerol) containing 1% (w/v) DTT for 15 minutes, and then incubated in equilibration buffer containing 2.5% (w/v) iodoacetamide for 15 minutes. Proteins were resolved in a 12.5% gel at 0.5 W/gel for 1 hour, then 17 W/gel for 3 hours using the Ettan DALT*six* gel running tank (GE Healthcare). Gels were either stained with Coomassie G-250 (10% (w/v) ammonium sulphate, 0.1% Coomassie (w/v) G-250, 3% (v/v) orthophosphoric acid, 20% (v/v) ethanol) or transferred onto Hybond-LFP (GE Healthcare) and immunoblotted with the Phospho-AKT Substrate (PAS) antibody (Supplementary Table  1) and Cy5 conjugated secondary antibody (GE Healthcare). Signals were detected by scanning with the Typhoon tri9100 (GE Healthcare). Total protein (Cy2) and PAS (Cy5) signals were overlayed using ImageQuant (GE Healthcare). Protein spots of interest were manually excised from the Coomassie G-250 stained gel and identified by mass spectrometry.

### 2.10. Mass Spectrometry

Protein samples are resolved by SDS-PAGE, Coomassie R-250 stained and proteins of interest excised, in-gel tryptic digested and identified using LC-ESI-MS/MS using an Agilent 1100 Series HPLC coupled to an Agilent LC/MSD Trap XCT Plus Mass Spectrometer fitted with an HPLC Chip cube (Agilent, Palo Alto, Calif). Mass spectrometry was performed by the Core Facility at Peter MacCallum Cancer Centre, utilising facilities at Bio21 RTF (Parkville, VIC, Australia). The data acquired were analysed by correlating the peptide masses obtained to predict peptide masses from proteins in the NCBI nonredundant database using the Mascot search engine (Matrix Science). 

## 3. Results

### 3.1. Relative Specific Activity of AKT Isoforms

**Figure 1:**
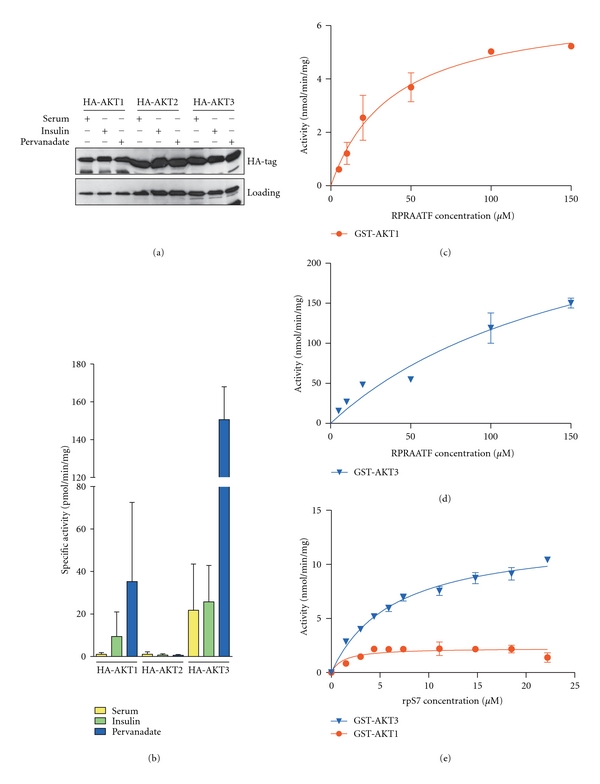
AKT3 is more active than AKT1 towards both the RPRAATF peptide and rpS7 protein substrate. HA-AKT isoforms were expressed in HEK293 cells, stimulated with 10% serum, 1 *μ*M insulin, or 0.1  *μ*M pervanadate and harvested into RLB. (a) expression of HA-AKT isoforms was detected by immunoblotting with the anti-HA antibody. (b) HA-AKT isoforms were immunoprecipitated from cleared protein lysates (100 *μ*g) and assayed towards the RPRAATF peptide for activity. Samples were assayed in duplicate. *n* = 1–3. Error bars: mean ± SD. GST-AKT1 and GST-AKT3 were expressed in HEK293 cells, stimulated with 0.1 *μ*M pervanadate, and harvested into RLB, then purified by GST-pull down and (c and d) assayed against increasing concentrations of the RPRAATF peptide or (e) rpS7. Data points were fitted to the Michaelis-Menten equation using GraphPad Prism version 5.00, GraphPad Software, San Diego, Calif, USA, http://www.graphpad.com/. *n* = 1, where samples were assayed in duplicate. Graph shows mean of duplicates.

Previously data from our laboratory using model peptide substrates revealed that AKT3 was ~15-fold more active than AKT1 which was ~10-fold more active than AKT2 [25]. To confirm that AKT2 was intrinsically considerably less active compared with AKT1 and 3 when expressed in HEK293 cells, HA-tagged versions of each isoform were immunopreciptated from HEK293 cells stimulated with serum, insulin, or pervanadate (Figures 1(a) and 1(b)). Under all conditions, AKT2 activity was minimal, despite robust expression. Thus, we have focused the present studies largely on AKT1 and 3.

To confirm that this difference in relative enzyme activity extended to the ability of the isoforms to phosphorylate a protein substrate, expressed GST-tagged AKT1 and 3 isoforms were purified from pervanadate-treated HEK293 cells and the kinetics of phosphorylation of the RPRAATF peptide [6, 31] compared to that with rpS7 protein. rpS7 has recently been identified as an *in vivo* substrate of the AKT signaling network [26], and we have confirmed this as a direct *in vitro* AKT substrate (Supplementary Figure  1). 

GST-AKT3 was ~47-fold more active than GST-AKT1 towards the RPRAATF peptide substrate, with their *V*_max_ values calculated as 315.90 ± 88.95 nmol/min/mg and 6.73 ± 0.69 nmol/min/mg, respectively (Figures 1(c) and 1(d)). GST-AKT3 was ~5-fold more active than GST-AKT1 in phosphorylating rpS7 with a *V*_max_ value of 12.67 ± 0.47 nmol/min/mg and 2.27 ± 0.20 nmol/min/mg, respectively (Figure 1(e)). While the differences in specific activity were marked, the fold change was considerably less with the protein compared to the synthetic peptide substrate. Furthermore, GST-AKT3 had a lower affinity for the rpS7 protein than GST-AKT1, with *K*_*m*_ values of 6.43 ± 0.62 *μ*M and 1.24 ± 0.63 *μ*M, respectively. Thus, despite the markedly higher *V*_max_ for AKT3, its elevated activity will be dependent on higher substrate concentrations than AKT1 meaning that the local concentrations of specific substrates may be critical in determining isoform-specific signaling.

### 3.2. Gain of Function Approach to Determine AKT Substrate-Specific Phosphorylation

**Figure 2:**
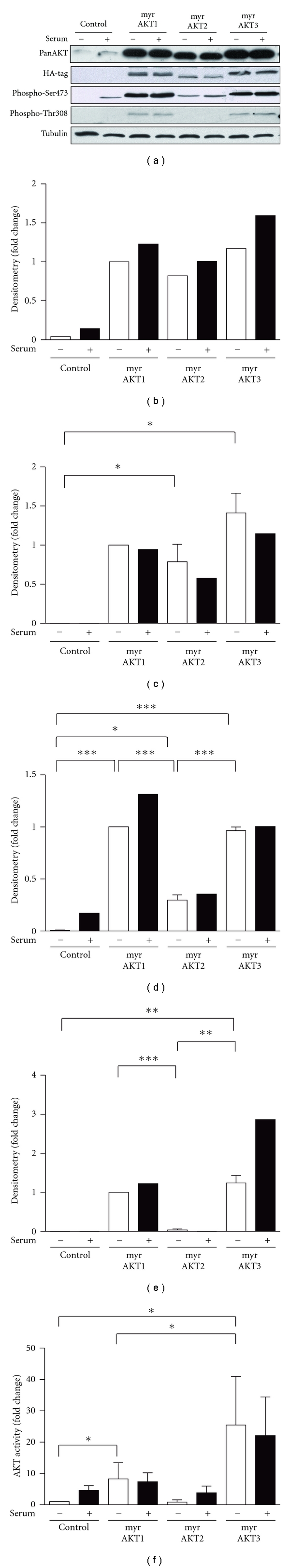
Comparison of AKT isoform-specific activation *in vivo *and activity *in vitro*. HEK293 cells were transfected with the pCDNA3 vector (control) or myrAKT isoforms, serum-starved for 24 hours, then stimulated with 10% serum for 20 minutes. (a) protein lysates (20–50 *μ*g) were separated by SDS-PAGE, transferred onto PVDF membrane, and immunoblotted. Western blots are representative of *n* = 1–5 experiments. Signals were quantified by densitometry using ImageJ 1.42 q (National Institutes of Health, USA), normalised to loading and expressed as fold change over myrAKT1 serum-starved samples. (b) panAKT. *n* = 1 (c) HA-tag. (d) phospho-Ser473. (e) phospho-Thr308. (c–e) Serum-starved samples: *n* = 4, stimulated samples: *n* = 1. Error bars: mean ± SD. (f) protein lysates (20 *μ*g) were incubated with the RPRAATF peptide substrate in the presence of [*γ*-^32^P]ATP at 30°C for 20 minutes to determine total AKT activity. Each sample was assayed in duplicate. Levels of AKT activity are represented as fold change over the serum-starved control sample. *n* = 2–6, Graph shows mean ± S.D. Statistical analysis was performed using the paired *t*-test (GraphPad Prism version 5.0, GraphPad Software, San Diego, Calif, USA). Paired *t*-test was not calculated between serum-starved control and myrAKT1 for (c and e) as the fold difference was the same for all blots quantified. *P* values >0.05 are not significant, *P* values 0.01 to 0.05 (*), *P* values 0.001 to 0.01 (**), and *P* values <0.001 (***).

**Figure 3:**
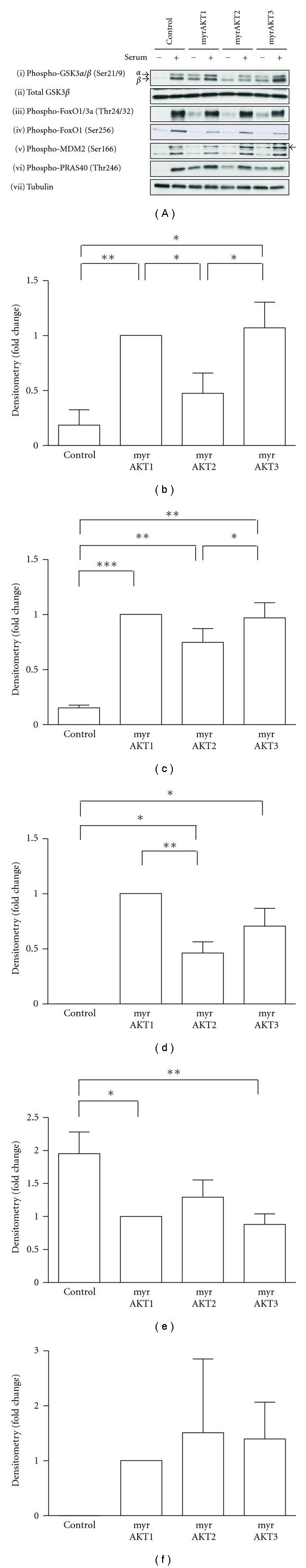
Differential isoform-specific signaling to direct AKT substrates *in vivo*. (a) Protein lysates (20–50 *μ*g) generated from HEK293 cells transfected with the pCDNA3 vector (control), or overexpressing HA-tagged myrAKT isoforms were resolved by SDS-PAGE, transferred onto membrane, and immunoblotted. Western blots are representative of *n* = 2–5 experiments. Signals from serum-starved samples were quantified by densitometry using ImageJ 1.42q (National Institutes of Health, USA), normalised to loading, and expressed as fold change over myrAKT1 serum-starved samples. (b) phospho-GSK3*α* (Ser21). *n* = 5. Error bars: mean ± SEM. (c) phospho-GSK3*β* (Ser9). *n* = 5. Error bars: mean ± SEM. (d) phospho-FoxO1/3a (Thr24/32). *n* = 5. Error bars: mean ± SEM. (e) phospho-FoxO1 (Ser256). *N* = 5. Error bars: mean ± SEM. (f) phosph-PRAS40 (Thr246). *n* = 2. Error bars: mean ± SD. Statistical analysis was performed using the paired *t*-test (GraphPad Prism version 5.0, GraphPad Software, San Diego, Calif, USA). Paired *t*-test was not calculated between serum-starved control and myrAKT1 for (d) as the fold difference was the same for all blots quantified. *P* values >0.05 are not significant, *P* values 0.01 to 0.05 (*), *P* values 0.001 to 0.01 (**), and *P* values < 0.001 (***).

In order to examine the potential outcomes of these different intrinsic enzyme properties on *in vivo* substrate phosphorylation, HA epitope tagged forms of constitutively activated N-terminal myristoylated (myr) AKT1, 2, and 3 were expressed in serum-starved or stimulated HEK293 cells (panAKT western blot, Figures 2(a) and 2(b)). Transfection conditions were optimised to achieve similar expression levels for each isoform as indicated by the anti-HA western blot (Figures 2(a) and 2(c)). Analysis of AKT regulatory phosphorylation sites revealed that enforced expression of each isoform resulted in constitutive phosphorylation at Ser473 (Figures 2(a) and 2(d)). Thr308 phosphorylation was also markedly increased in myrAKT1 and myrAKT3 transfected cells (Figures 2(a) and 2(e)), consistent with elevated total AKT activity compared with cells expressing myrAKT2. Direct kinase assays confirmed that myrAKT1 and 3 exhibit constitutive activity (Figure 2(f)). To examine the *in vivo* effects of enforced expression of these specific isoforms, we took a targeted substrate approach, focusing on known direct AKT substrates involved in key cellular processes: proliferation (GSK3*α* and GSK3*β*); survival (FoxO1, FoxO3a and MDM2); growth (PRAS40) (Figure 3). 

#### 3.2.1. Effect of Enforced AKT Isoform Expression on Proliferation: GSK3*α* and GSK3*β*

Glycogen synthase kinase 3 (GSK3) is a negative regulator of cell proliferation [4, 32, 33] and has been implicated in various diseases including diabetes, alzheimer's, and cancer [34]. GSK3 is expressed as two isoforms, GSK3*α* and GSK3*β*, in similar tissues [35], and their activity is inhibited by AKT which phosphorylates the regulatory site Ser21 and Ser9, respectively [32, 36]. Overexpression of myrAKT1 and 3, but not myrAKT2, significantly increased GSK3*α* (Ser21) phosphorylation under serum-starved conditions (Figures 3(a)(i), and 3(b)). However, all three myrAKT isoforms significantly increased GSK3 (Ser9) phosphorylation under serum-starved conditions (Figures 3(a)(i), and 3(c)), suggesting that AKT2 specifically targeted GSK3*β* over GSK3*α* despite its apparent extremely low peptide phosphorylating activity. The data presented here suggests that constitutive AKT1 and 3 but not AKT2 activity are sufficient to phosphorylate GSK3*α*, while all three isoforms are sufficient in phosphorylating GSK3*β*.

#### 3.2.2. Effect of Enforced AKT Isoform Expression on Survival: FoxO1, FoxO3, and MDM2

One mechanism by which AKT modulates cell survival involves phosphorylation and thus inactivation of the FoxO family of transcription factors which regulate the expression of various genes associated with apoptosis, the cell cycle, and DNA repair [37]. AKT directly phosphorylates FoxO1 at residues Thr24 and Ser256, and FoxO3a at Thr32 [37–40], however, other kinases are also capable of FoxO protein phosphorylation [37–40]. 

In this study, FoxO1 (Thr24) and FoxO3a (Thr32) were robustly phosphorylated in response to serum (Figure 3(a)(iii)). In serum-starved conditions, the overexpression of all three constitutively active AKT isoforms (Figures 3(a)(iii), and 3(d)) modestly increased FoxO1/3a (Thr24/32) phosphorylation with myrAKT1 and 3 being more potent than myrAKT2. This suggests that all isoforms are sufficient to phosphorylate FoxO1/3a at residues Thr24 and 32, respectively. However, in all three cases this was not additive with serum treatment (Figure 3(a)(iii)) indicating that serum stimulation causes maximal phosphorylation at these sites. Interestingly, none of the constitutively active myrAKT isoforms was sufficient to induce FoxO1 (Ser256) phosphorylation in serum-starved conditions (Figures 3(a)(iv), and 3(e)). Indeed, serum induced phosphorylation of FoxO1 at Ser256 trended lower upon expression of constitutively active AKT isoforms.

AKT also regulates cell survival by phosphorylating murine double minute 2 (MDM2) at residue Ser166, which results in its translocation into the nucleus and binding to p53, a tumour suppressor that acts as a transcription factor to express proapoptotic genes. The p53/MDM2 complex is then sequestered to the cytoplasm for proteasomal degradation [4, 41, 42]. Overexpression of all myrAKT isoforms was sufficient to induce phosphorylation under serum-starved conditions (Figure 3(a)(v), top band indicated by arrow), thus suggesting that all isoforms are sufficient to phosphorylate MDM2 at Ser166. However, this phosphorylation was not additive with serum when compared to the control. The differences in the signal to noise between individual western blots made statistical analysis of MDM2 phosphorylation from multiple gels impractical. However, as consistent trends were observed, a representative blot is presented.

#### 3.2.3. Effect of Enforced AKT Isoform Expression on Growth: PRAS40

AKT signals to mTORC1 via two converging pathways to regulate cell growth. One pathway involves the proline rich AKT substrate of 40 kDa (PRAS40) and the other the tuberous sclerosis complex 2 (TSC2) [43–45]. Specifically, PRAS40 inhibits Ras homolog enriched brain-(Rheb)-dependent mTORC1 activity by binding to the raptor subunit of mTORC1, and this is relieved upon its phosphorylation at Thr246 by AKT [46]. Overexpression of each of the constitutively active AKT isoforms resulted in robust PRAS40 (Thr246) phosphorylation in serum-starved conditions (Figures 3(a)(vi), and 3(f)). As observed for FoxO1/3a, this phosphorylation was not additive upon serum stimulation, nor was any differential isoform specificity observed.

### 3.3. Effect of Enforced AKT Signaling down the mTORC1 Pathway

**Figure 4:**
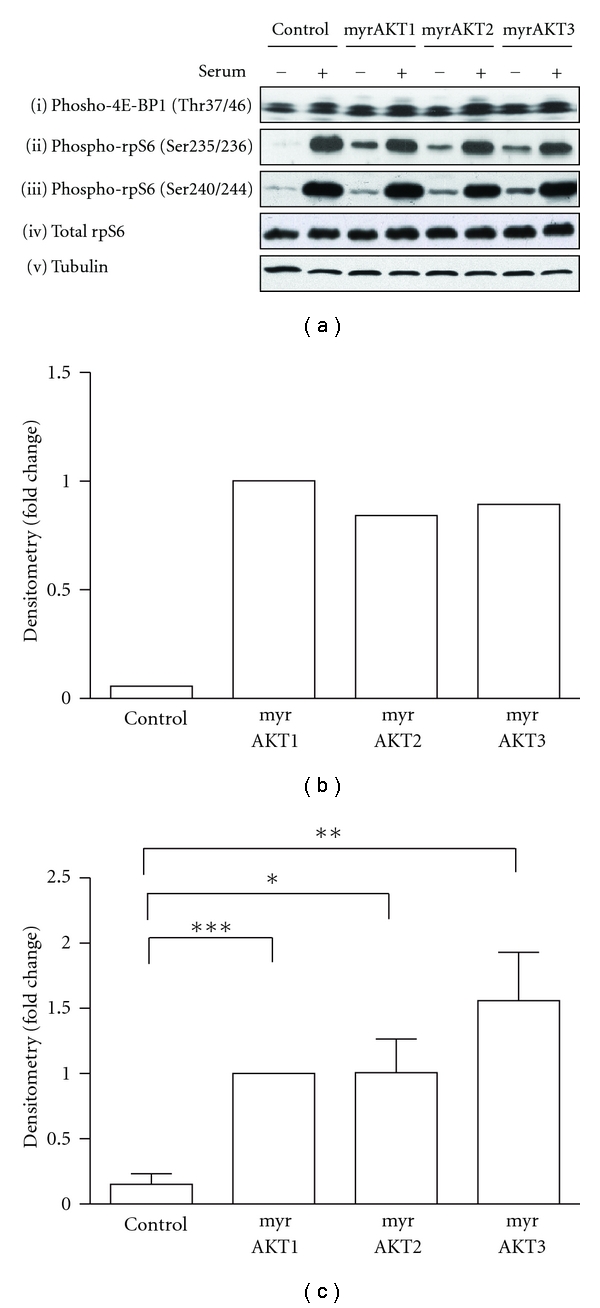
Expression of all three AKT isoforms is sufficient for signaling down the mTORC1 pathway. (a) Protein lysates (20–25 *μ*g) generated from HEK293 cells transfected with the pCDNA3 vector (control), or overexpressing HA-tagged myrAKT isoforms were resolved by SDS-PAGE, transferred onto membrane, and immunoblotted. Western blots are representative of *n* = 1  –5 experiments. Signals from serum-starved samples were quantified by densitometry and normalised to loading and expressed as fold change over myrAKT1 serum-starved samples. (b) phospho-rpS6 (Ser235/236). *n* = 1. (c) phospho-rpS6 (240/244). *n* = 5. Error bars: mean ± SEM. Statistical analysis was performed using the paired *t*-test (GraphPad Prism version 5.0, GraphPad Software, San Diego, Calif, USA). *P* values >0.05 are not significant, *P* values 0.01 to 0.05 (*), *P* values 0.001 to 0.01 (**), and *P* values < 0.001 (***).

AKT is known to mediate cell growth by signaling through the mTORC1 pathway primarily due to mTORC1 phosphorylation of initiation factor 4E-Binding Protein 1 (4E-BP1) and activation of S6 Kinase 1 (S6K1) that phosphorylates ribosomal protein S6 (rpS6) [47]. mTORC1 also activates rDNA transcription [48]. These signals converge to promote ribosomal biogenesis and function [49]. Thus in this study 4E-BP1 and rpS6 were used as readouts of AKT signaling down the mTORC1-growth signaling pathway (Figure 4).

#### 3.3.1. Effect of Enforced AKT Isoform Expression on 4E-BP1

The function and activity of 4E-BP1 is modulated by phosphorylation at multiple residues [50]. The 4E-BP1 residues Thr37 and Thr46 are priming sites that require phosphorylation by mTORC1 before other key residues are available to kinases such as ERK2 and JNK [50, 51]. In serum-starved conditions overexpression of all myrAKT isoforms modestly increased 4E-BP1 (Thr37/46) phosphorylation when compared to control (Figure 4(a)(i)). Again, this phosphorylation was not additive upon serum stimulation, nor was any differential isoform specificity observed. As for MDM2, the differences in the signal to noise between individual western blots made statistical analysis of 4E-BP1 (Thr37/46) phosphorylation from multiple gels impractical. However, as consistent trends were observed, a representative blot is presented.

#### 3.3.2. Effect of Enforced AKT Isoform Expression on Ribosomal Protein S6

Activation of the mTORC1/S6K1 pathway results in the phosphorylation of rpS6 at Ser235, Ser236, Ser240, and Ser244 [52, 53]. In serum-starved cells, overexpression of all three myrAKT isoforms increased rpS6 Ser235/236 and Ser240/244 phosphorylation when compared to the control (Figures 4(a)(ii)(iii), 4(b) and 4(c)), although this was considerably less than the phosphorylation observed in response to serum stimulation. Like 4E-BP1, no further elevation in rpS6 Ser235/236 and Ser240/244 phosphorylation was observed upon serum stimulation. 

In conclusion, these experiments reveal that despite the enforced expression of AKT isoforms with very different intrinsic kinase activities towards defined peptide and protein substrate, the resulting *in vivo* phosphorylation patterns of known substrates is largely isoform independent. Some subtle differences were identified including the lack of phosphorylation of GSK3*α* by myrAKT2 and its reduced phosphorylation of FoxO1/3a (Thr24/32).

### 3.4. Loss of Function Approaches to Determine AKT Substrate Specific Phosphorylation

**Figure 5:**
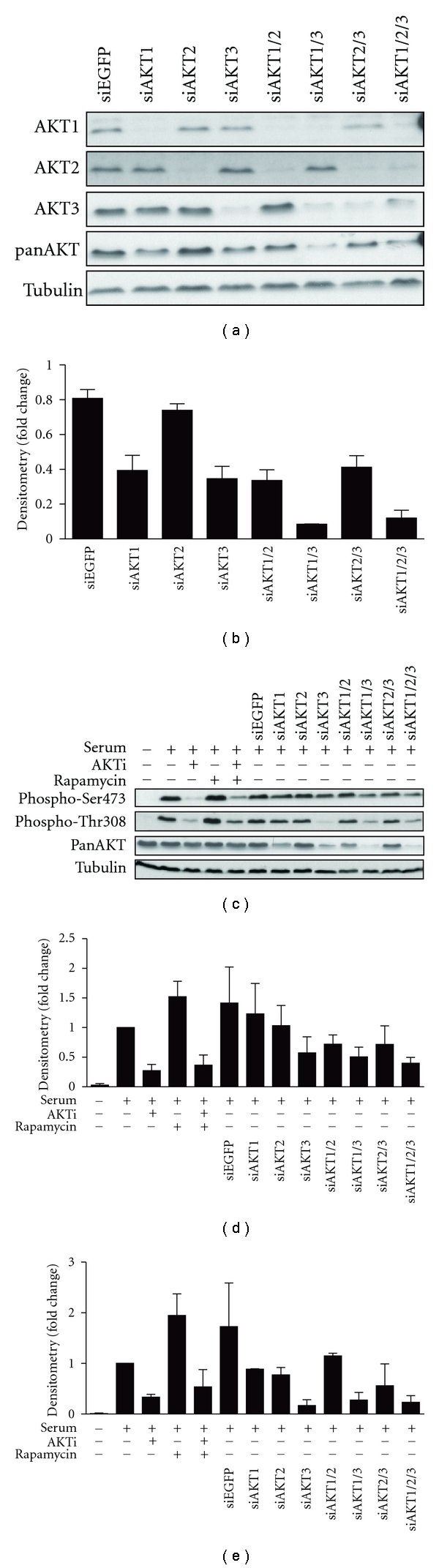
Specific knockdown of endogenous AKT isoforms. HEK293 cells were serum-starved for 24 hours and pretreated with either 5 *μ*M AKTi, 20 nM rapamycin, or both for 30 minutes prior to stimulation with 10% serum for 20 minutes and harvesting into RLB. Endogenous AKT expression was knocked down, either individually or simultaneously, with 25 nM of siRNAs towards specific AKT isoforms and harvested into RLB. siEGFP was used as the control. Protein lysates (20–25 *μ*g) were separated by SDS-PAGE, transferred onto PVDF membrane, and immunoblotted. Western blots are representative of *n* = 3 experiments. Western blot signals were quantified by densitometry, normalised to loading and expressed as fold change over the serum-stimulated control. (a) Specificity of isoform-specific knockdown and their effects on total AKT expression were analysed by immunoblotting with isoform-specific and panAKT antibodies. (b) panAKT. *n* = 3. Error bars: mean ± SEM. (c) Total AKT activation levels were analysed by immunoblotting with phospho-Ser473 and phospho-Thr308 antibodies. (d) Phospho-Ser473. *n* = 3. Error bars: mean ± SEM. (e) Phospho-Thr308. *n* = 2, Error bars: mean ± SD.

**Figure 6:**
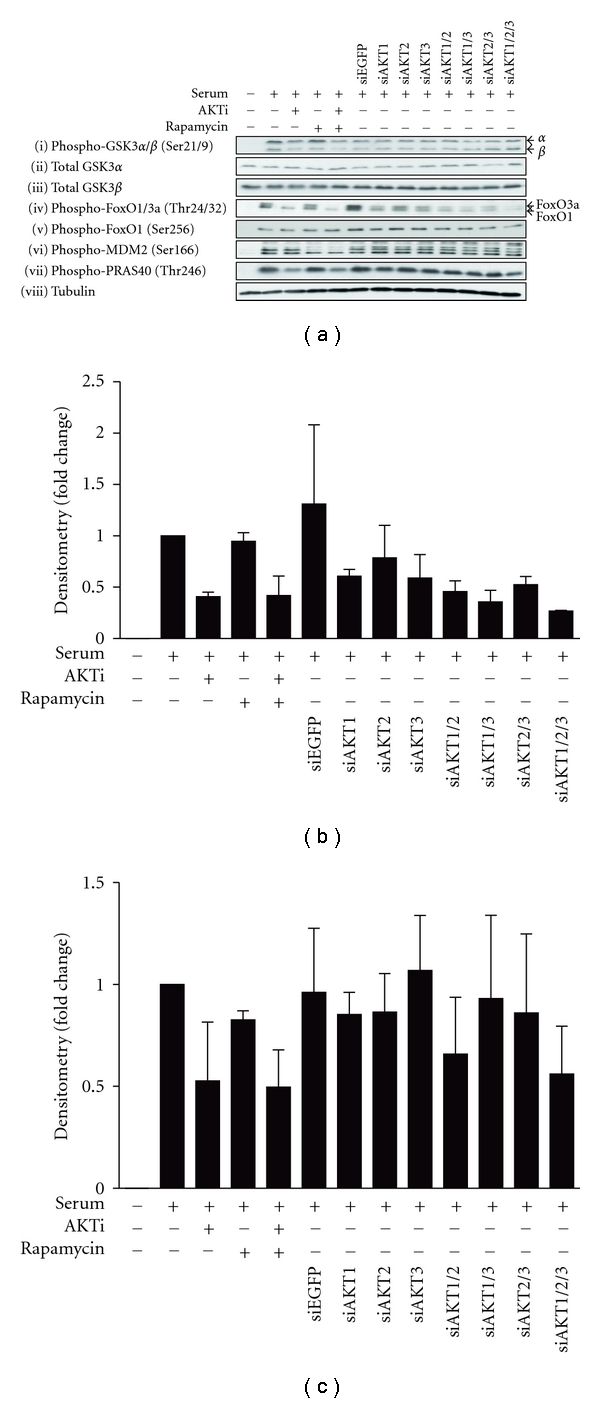
Dependence of AKT isoform-specific expression for signaling down cell proliferation, survival, and growth pathways. (a) HEK293 cells were serum-starved for 24 hours before pretreatment with 5 *μ*M AKTi, 20 nM rapamycin or both for 30 minutes prior to stimulation with 10% serum and harvested into RLB. Expression of endogenous AKT isoforms were knocked down in HEK293 cells, either individually or simultaneously, with 25 nM of siRNAs towards specific AKT isoforms and harvested into RLB. siEGFP was used as the control. Protein lysates (20–25 *μ*g) were separated by SDS-PAGE, transferred onto PVDF membrane, and immunoblotted. Western blots are representative of *n* = 3 experiments. Intensity of western blot signals were quantified by densitometry, normalised to loading, and expressed as fold change over the serum-stimulated control. (b) phospho-FoxO1/3a (Thr24/32). *n* = 2. Error bars: mean ± SD. (c) phospho-PRAS40 (Thr246). *n* = 2. Error bars: mean ± SD.

To further assess potential differences in the requirement for specific isoforms for substrate phosphorylation we used siRNA approaches to knockdown the expression of each isoform individually and in combination (Figures 5 and 6). Western blot analysis using isoform-specific antibodies showed that the siRNAs used specifically targeted their respective AKT isoform (Figure 5(a)). Moreover, panAKT western blot analysis indicated that of the three isoforms, the individual knockdown of AKT1 and AKT3 most robustly reduced total AKT expression (Figures 5(a) and 5(b)), thus suggesting that these two isoforms may be more abundantly expressed in HEK293 cells. Moreover, when both AKT1 and AKT3 expression were reduced, an additive reduction in endogenous AKT expression was demonstrated (Figures 5(a) and 5(b)).

The contribution of each AKT isoform to total AKT activity *in vivo* was also analysed by examining the levels of phosphorylation at the two regulatory residues Ser473 and Thr308 (Figures 5(c)–5(e)). It should be noted that combined knockdown of all isoforms did not fully ablate AKT expression or phosphorylation but did reduce the levels of active kinase to a similar level to the AKT inhibitor (AKTi), which we have shown to inhibit all AKT isoforms in HEK293 cells when used at 5 *μ*M (Chan et al. [54]). 

As with the overexpression studies we took a targeted approach focusing on AKT substrates involved in key cellular processes: proliferation (GSK3*α* and GSK3*β*); survival (FoxO1, FoxO3a and MDM2); growth (PRAS40). However, many direct AKT substrates are also phosphorylated by other kinases; for example, GSK3*β* and MDM2 are also substrates of S6K1, a downstream kinase of mTORC1 [55, 56]. Thus, to delineate direct AKT substrates from mTORC1-mediated signaling, cells were treated with specific inhibitors of AKT (AKTi) and/or mTORC1 (rapamycin) (Figure 6). 

#### 3.4.1. Effect of AKT Isoform Knockdown on Proliferation: GSK3*α* and GSK3*β*

Specific reduction in AKT isoform expression individually or in combination did not modulate the level of phosphorylated or total GSK3*α* (Ser21) or GSK3*β* (Ser9) (Figure 6(a)(i)–(iii)). In fact, complete inhibition of AKT activity with AKTi only marginally reduced GSK3 phosphorylation (Figure 6(a)(i)) despite the results showing their dependence on AKT expression in the overexpression studies (Figure 3(a)(i)). This may be due to other kinases such as PKA and S6K1 that have been shown to also play an active role in GSK3*α* (Ser21) and GSK3 (Ser9) phosphorylation [57]. In summary, under the conditions tested, AKT is sufficient but not necessary for phosphorylation of GSK3*α* (Ser21) and GSK3 (Ser9).

#### 3.4.2. Effect of AKT Isoform Knockdown on Survival: FoxO1, FoxO3, and MDM2

Phosphorylation of FoxO1/3a (Thr24/32) was markedly reduced upon knockdown of each of the three AKT isoforms with AKT1 and 3 being more potent than AKT2 (Figures 6(a)(iv), and 6(b)). This is consistent with overexpression studies where myrAKT1 and 3 were shown to phosphorylate FoxO1/3a (Thr24/32) more intensely (Figures 3(a)(iii), and 3(d)). Moreover, an additive reduction in FoxO1/3a (Thr24/32) phosphorylation was demonstrated upon the knockdown in expression of all three AKT isoforms (Figures 6(a)(iv), and 6(b)). When combined with the AKT overexpression studies we conclude that all AKT isoforms are both sufficient and necessary for the phosphorylation of FoxO1/3a at Thr24 and Thr32, respectively. In contrast knockdown of all AKT isoforms together had no effect on FoxO1 at Ser256 (Figure 6(a)(v)). This is consistent with the data of Brognard et al. [58] who also failed to observe altered FoxO1 (Ser256) phosphorylation upon the knockdown of AKT isoform-specific expression in H157 cells, a non-small-cell lung cancer cell line. Additionally, treatment with AKTi did not alter FoxO1 phosphorylation at Ser256 (Figure 6(a)(v)). This is consistent with the lack of effect of overexpressing AKT on this substrate (Figures 3(a)(iv), and 3(e)). Taken together, these results suggest that in HEK293 cells, AKT may mediate cell survival by phosphorylating FoxO1/3a at Thr24/32 but not Ser256. 

Phosphorylation of MDM2 (Ser166) was not altered upon the individual or compound knockdown of the AKT isoforms (Figure 6(a)(vi)). This suggests that AKT expression is not necessary for MDM2 Ser166 phosphorylation, despite overexpression of AKT being sufficient to increase its phosphorylation (Figure 3(a)(v)). Moreover, MDM2 phosphorylation at Ser166 was not reduced upon treatment with AKTi (Figure 6(a)(vi)). This is in contrast to treatment with the mTORC1 inhibitor rapamycin, which had a dramatic effect on MDM2 (Ser166) phosphorylation (Figure 6(a)(vi)). This suggests that under these conditions, AKT does not regulate MDM2 (Ser166) phosphorylation while mTORC1 does and that mTORC1 must be activated in an AKT-independent manner [59–61].

#### 3.4.3. Effect of AKT Isoform Knockdown on Growth: PRAS40

Phosphorylation of PRAS40 (Thr246) was not significantly modulated by knockdown of any individual AKT isoform (Figures 6(a)(vii), and 6(c)) although there was a subtle reduction when RNAi to all three AKT isoforms were combined. Moreover, AKTi treatment, but not rapamycin, reduced PRAS40 (Thr246) phosphorylation (Figures 6(a)(vii), and 6(c)). In combination with the above data this would suggest that AKT is both sufficient and necessary for PRAS40 (Thr246) phosphorylation; however the AKT isoforms are functionally complementary with respect to this substrate, with knockdown of all giving additive inhibition.

#### 3.4.4. Effect of AKT Isoform Knockdown on mTORC1 Signaling Pathway

**Figure 7:**
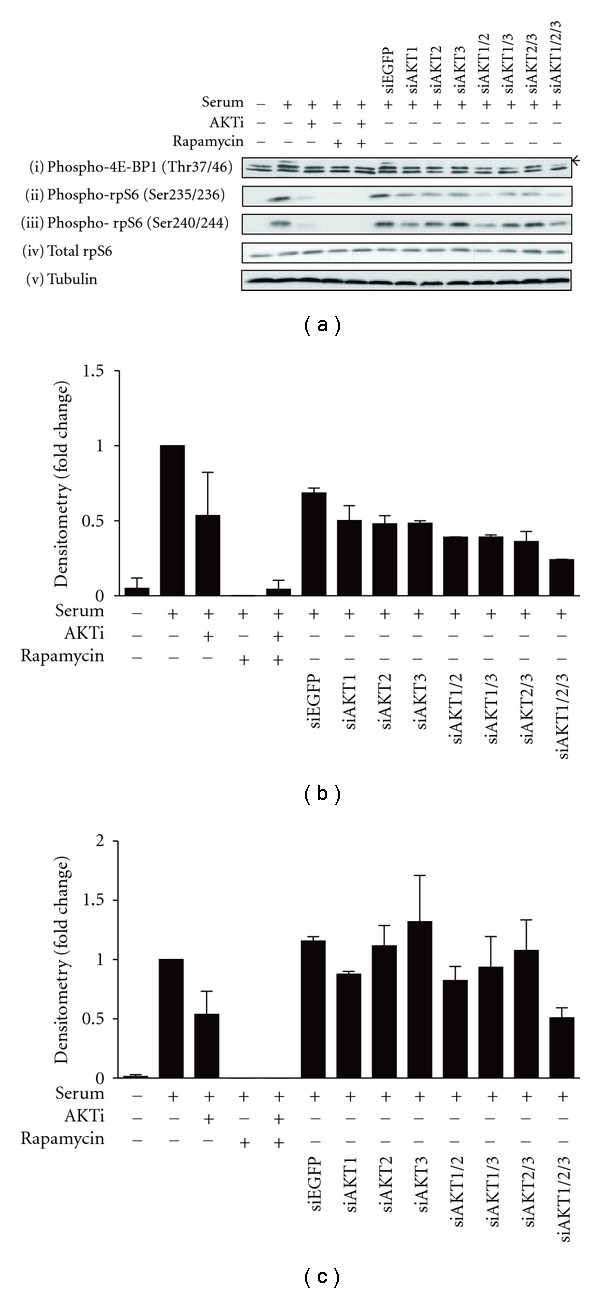
Effect of AKT expression on activation of the mTORC1 pathway. (a) HEK293 cells were serum-starved for 24 hours before treatment with 5 *μ*M AKTi, 20 nM rapamycin or both for 30 minutes prior to stimulation with 10% FBS and harvested into RLB. Expression of endogenous AKT isoforms were knocked down in HEK293 cells, either individually or simultaneously, with 25 nM of siRNAs towards specific AKT isoforms, and harvested into RLB. siEGFP was used as the control. Protein lysates (20–25 *μ*g) were separated by SDS-PAGE, transferred onto PVDF membrane, and immunoblotted. The arrow indicates the hyperphosphorylated band of phospho-4E-BP1. Western blots are representative of *n* = 3 experiments. Intensity of western blot signals was quantified by densitometry, normalised to loading and expressed as fold change over the serum-stimulated control. (b) Phospho-rpS6 (Ser235/236). *n* = 2. Error bars: mean ± SD. (c) Phospho-rpS6 (Ser240/244). *n* = 3. Error bars: mean ± SEM.

Consistent with the modest increase in 4E-BP1 (Thr37/46) phosphorylation induced by all constitutively active AKT isoforms (Figure 4(a)), knockdown of each individual AKT isoform reduced 4E-BP1 phosphorylation as evidenced by disappearance of the lowest mobility species (Figure 7(a)(i), indicated by an arrow). Furthermore, phosphorylation of the Ser235/236 site on rpS6 (Figures 7(a)(ii), and 7(b)) was also reduced upon knockdown of each individual AKT isoform. In contrast, reduction of phosphorylation of Ser240/244 in rpS6 required knockdown of AKT1 and was optimal with all three (Figures 7(a)(iii), and 7(c)) raising the possibility that AKT1 exhibits some nonredundant signaling to rpS6 phosphorylation. Interestingly for both of these substrates all three of these phosphorylation sites were robustly reduced with either AKTi treatment or rapamycin (Figure 7(a)(i–iii)), consistent with the importance of AKT in the regulation of mTORC1 function which functions upstream of both 4E-BP1 and rpS6.

### 3.5. Differences in Signaling between AKT Isoforms Are Subtle *In Vivo*

**Figure 8:**
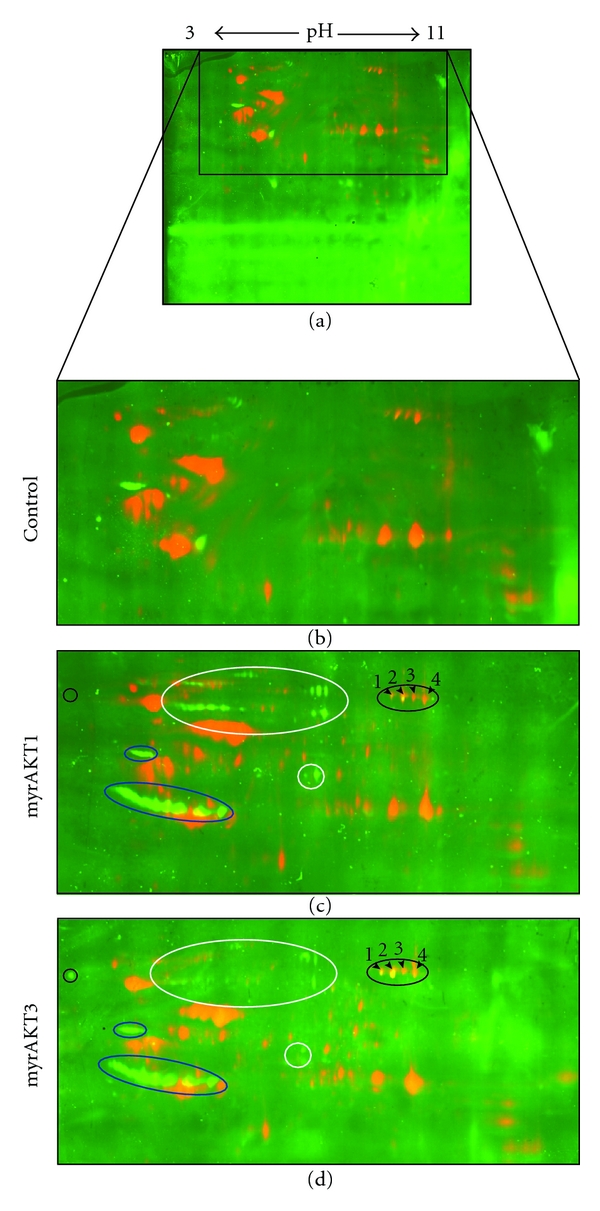
Identification of eEF2 as a potential AKT substrate. HEK293 cells transfected with the pCDNA3 vector (control) or expressing similar levels of myrAKT1 or myrAKT3 were serum-starved for 24 hours prior to harvesting into RLB. Samples were processed in duplicate. Cy2 labelled protein samples (250 *μ*g) were loaded onto 18 cm broad range IPG strips with a nonlinear pH range of 3–11, focused and resolved by SDS-PAGE. After 2DGE, gels were transferred onto Hybond-LFP membrane and then immunoblotted with the PAS antibody and Cy5-conjugated secondary antibody. Membranes were scanned using the Typhoon trio9100 for both Cy2 and Cy5 signals. Cy2 and Cy5 signals were overlayed using ImageQuant (GE Healthcare). Cy2 (total protein) signals are represented in red. Cy5 (PAS) signals are represented in green. Overlayed signals are represented in yellow. (a) control. (b–d) enlarged region of membrane containing control, myrAKT1 or myrAKT3 samples, respectively. Proteins more efficiently phosphorylated by myrAKT1 are circled in white, by myrAKT3 are circled in black, and with equal efficiencies for both are circled in blue. The four protein spots (spots 1–4) were excised from the myrAKT1 Coomassie R-250 stained gel (Supplementary Figure  2) and identified as eEF2 by mass spectrometry analysis (Supplementary Figure  3).  *n* = 1.

These data demonstrate dramatic differences in peptide kinetics for the three AKT isoforms, which were less marked when using a protein substrate *in vitro* (Figures 1(c)–1(e)). However regulation of the level of phosphorylation for some known *in vivo* substrates, whether by overexpression or specific knockdown of individual AKT isoforms, was extremely subtle. Only GSK3*α* (Ser21) and FoxO1/3a (Thr24/32) exhibited clear differential regulation by the AKT isoforms. Thus we took a third approach, to identify potential AKT isoform-specific substrates by performing an unbiased screen on cells overexpressing myrAKT1 or myrAKT3 and utilizing 2DGE combined with western blot analysis with the phospho-AKT substrate (PAS) antibody that detects phosphorylation at AKT-specific motifs [62]. Consistent with our studies of validated substrates, the patterns of phosphorylation of more than 30 AKT regulated PAS signals were similar (Figure 8). However, some differences were also observed. A group of proteins were found to be more efficiently phosphorylated by myrAKT1 compared to myrAKT3 (Figures 8(c) and 8(d), white circle) while 5 phosphoproteins 4 of which were seemingly related, were more efficiently phosphorylated by myrAKT3 than myrAKT1 (Figures 8(c) and 8(d), black circle). Of the 5 spots more efficiently phosphorylated by myrAKT3, four overlapped with Coomassie G-250 stained spots (Supplementary Figure  2(c)), and these were excised, digested, and subjected to mass spectrometry analysis. All four spots were identified as eukaryotic translation elongation factor 2 (eEF2, Supplementary Figure  3). The potential and significance of AKT mediated regulation of eEF2 remains to be elucidated. Thus, in general the patterns of phosphorylation observed using the 2DGE approach were similar for the two AKT isoforms, although some subtle isoform-specific differences were observed, consistent with the findings obtained above for GSK3 and FoxO1/3a phosphorylation (Figure 3). Thus, while relative expression levels are likely to underlie the major isoform-specific signaling observed at least in HEK293 cells, differences in intrinsic enzyme activity may contribute to subtle modifications of the differential effects of AKT on the regulation of a range of cellular processes.

## 4. Discussion

AKT consists of a family of three homologous isoforms, AKT1, 2, and 3, whose role in maintaining cellular homeostasis is thought to be pivotal in regulating processes such as cell survival, growth, proliferation, angiogenesis, and metabolism [3]. While much is known about the complex regulation of this pathway, many questions remain unanswered. Though the substrates that mediate some of its pleiotropic effects have been characterised, many critical substrates remain to be identified [3]. Furthermore, studies involving single and double knockout mice suggest that the AKT isoforms may differentially signal to produce distinct phenotypes [11–17, 63–65]. The basis of this differential signaling and its impact on determining the roles of the individual isoforms in regulating specific cellular functions remain unclear. 

Recently, potential AKT isoform-specific substrates have come to light, providing one clear mechanism for differential signaling; using loss-of-function studies, Brognard et al. [58] showed that TSC2 (Ser939 and Thr1462) phosphorylation was dependent on AKT1 and AKT2, but not AKT3, expression. Moreover, they showed that p27 (Thr157) phosphorylation was specifically dependent on AKT3 expression. Additionally GSK3*α* (Ser21) and MDM2 (Ser166) phosphorylation was shown to be exclusively dependent on AKT2 expression.

Here we have addressed the question of whether isoform-specific signaling was due to differences in the intrinsic activity of the isoforms. Previous reports have shown that AKT3 is the most active isoform towards a range of peptide substrates [21, 24, 25]. We confirmed that AKT3 had the highest specific activity compared to AKT1 and AKT2, phosphorylating the RPRAATF synthetic peptide with~47-times the specific activity of AKT1. Importantly, we showed that this hierarchy of activity was conserved for a protein substrate (rpS7), but the difference in activity was reduced to 5-fold. These data indicated that the differences in intrinsic kinase activity towards protein substrates may be more modest than first thought and potentially of less impact *in vivo*. Indeed, this concept is reinforced by the observation that AKT3 had a 5-fold lower affinity than AKT1 towards the rpS7 protein meaning at limiting substrate concentrations, the activity of the isoforms may be very similar. Similar differences in the *K*_*m*_ for peptide phosphorylation were observed previously [25]. Alternatively, it is possible that colocalization of AKT3 with important substrates will facilitate local concentrations required for maximum AKT3 activity and provide a measure for isoform-specific signaling not evident in the cytoplasm. Such sites may include the translational apparatus or the nucleolus where ribosomal proteins, such as rpS7 and ribosomal RNA are assembled into ribosomes. Supporting this hypothesis is the recent finding that AKT3 localises to the nucleus in a range of cell lines including HEK293 [20]. 

To further examine the potential for isoform-specific phosphorylation of substrates *in vivo* we determined the effect of enforced expression of constitutively active isoforms on the phosphorylation of key validated substrates, GSK3*α* and GSK3*β*, FoxO1/3a, MDM2, and PRAS40. These observations were complemented by loss-of-function studies using isoform-specific siRNA reagents. Regulation of the level of phosphorylation of these *in vivo* substrates, whether by overexpression or specific knockdown of individual AKT isoforms, was extremely subtle, indicating that relative expression levels are likely to underlie the major isoform-specific signaling observed at least in HEK293 cells. Expression of each AKT isoform contributed to the phosphorylation of FoxO1 at residue Thr24, but not at Ser256 (Figure 6(a)(iv)(v), and 3(d) and 3(e)) consistent with previous findings in H157, a non-small-cell lung cancer cell line [58]. In addition, we showed isoform-specific differences in the phosphorylation of FoxO1/3a (Thr24/32). Overexpression of myrAKT1 and 3 resulted in a greater level of FoxO1/3a (Thr24/32) phosphorylation when compared to myrAKT2 (Figure 3(a)(iii), and 3(d)). Consistent with these findings, the knockdown of AKT1 and AKT3 expression individually reduced the level of FoxO1/3a (Thr24/32) to a greater extent than AKT2 (Figure 6(a)(iv), and 6(b)). This indicates that the isoforms might vary in the strength or efficiency to phosphorylate FoxO1/3a (Thr24/32), thus resulting in different abilities to regulate cell survival and proliferation.

Taken together, these data indicate that despite exhibiting very different intrinsic enzyme activity, *in vivo* phosphorylation patterns induced by the activation of specific AKT isoforms are very similar when expressed at similar levels in a given cell type. These findings imply that differential expression, activation, or localization of the isoforms may play more dominant roles in determining isoform-specific functions. However, differences in intrinsic enzyme activity may contribute to subtle modifications of the differential effects of AKT on the regulation of a range of cellular processes, including glucose metabolism and cell proliferation (via GSK3) and cell survival (via FoxO1/3a). It is important to note that the basis of isoform-specific signaling is likely to vary depending on the type of cell or tissue. Certainly, recent studies suggest that the substrates targeted by different AKT isoforms can vary depending on cellular and tissue context [12]. 

AKT isoforms have been shown to localise to different subcellular compartments in a cell-line-specific manner as described above [19, 20]. It remains possible that overexpression of individual isoforms may subvert signal specificity by changing/overwhelming the specificity of AKT subcellular localization. Moreover, the AKT isoforms are expressed in various tissues at different levels. AKT1 is ubiquitously expressed, while AKT2 is predominantly expressed in brown fat and the heart [16], correlating with the role of AKT2 in diabetes and glucose metabolism. AKT3 was most abundantly expressed in the brain [16] correlating with its role in attaining normal brain size. Furthermore, there is growing evidence that the differential expression of specific AKT isoforms is associated with individual tumour types. AKT1 activity is frequently elevated in breast and prostate cancers [66] while AKT2 has been shown to be upregulated in pancreatic and ovarian carcinomas [67, 68]. We have shown that AKT3 is upregulated in 20% of ovarian cancers [25]. Thus, it is also possible that, while our results reveal only subtle differences in isoform-specific signaling in epithelial cells, this may vary in other cell types. Further experiments will be required to explore this possibility using a panel of primary human cell types.

##  Conflict of Interests

The authors declare that there is no conflict of interests.

## Supplementary Material

Supplementary material containing Supplementary Material and Methods, one Supplementary Table of the antibodies used in the study and three Supplementary Figures is available online at dois 10.4061/2011/720985.

## Abbreviations

4E-BP1: 4E-binding protein 1
FoxO: Forkhead box O
eEF2: Eukaryotic translation elongation factor 2
HEK293: Human embryonic kidney cell line
MDM2: Murine double minute 2
MS: Mass spectrometry
mTOR: Mammalian target of rapamycin
mTORC1: mTOR Complex 1
mTORC2: mTOR Complex 2
myr: Myristoylated
PAS: Phospho-AKT Substrate
PDK1: Phosphoinositide dependent kinase 1
PRAS40: Proline-rich AKT substrate of 40 kDa
rpS6: Ribosomal protein S6
rpS7: Ribosomal protein S7
S6K1: S6 Kinase 1
siRNAs: Small interfering ribonucleic acids.
