# Supplementary material for: Relative Expression Levels Rather Than Specific Activity Plays the Major Role in Determining In Vivo AKT Isoform Substrate Specificity
Source: Enzyme Res. 2011 Aug 22;2011:720985. doi: 10.4061/2011/720985 (PMC3160084; doi:10.4061/2011/720985)
Supplement: Supplementary file 1 — Supplementary material containing Supplementary Material and Methods, one Supplementary Table of the antibodies used in the study and three Supplementary Figures is available online at dois 10.4061/2011/720985. [file 720985.f1.pdf]

Supplementary data

Supplementary materials and methods

Screening for AKT substrates in 40S ribosomes

40S ribosomes (purified from rat liver as described in [66]) were incubated with purified GST-AKT1 for 30 min at 30°C in a reaction mixture (4.25 μl dilution buffer (50 mM MOPS, pH 7.2, 10 mM pNPP, 10 mM $MgCl_2$, 0.1 % Triton X-100) and 7.5 μCi [γ-^{32}P]ATP) with a final volume of 20 μl. Ribosomal proteins were resolved by SDS-PAGE, the gel stained with Coomassie R-250 and dried between cellophane. Phosphorylation signals were detected by exposure to film.

Supplementary Table I. Antibodies.

**Primary Antibody**	**Dilution/ concentration used**	**Source**	**Catalogue Number**
HA-tag (12CA5)	1:2000	Pearson Laboratory	-
panAKT	1:1000	CST	9272
phospho-Ser473	1:2000	CST	2971
phospho-Thr308	1:1000	CST	4056
AKT1	1:1000	CST	2967
AKT2	1:1000	CST	2964
AKT3	1:2000	Upstate	07-383
phospho-GSK3α/β (Ser21/9)	1:2000	CST	9331
phospho-GSK3β (Ser9)	1:2000	CST	9336
Total GSK3α	1:2000	CST	9338
Total GSK3β	1:2000	CST	9315
phospho-FoxO1/3a (Thr24/32)	1:2000	CST	9464
phospho-FoxO1 (Ser256)	1:2000	CST	9461
phospho-MDM2 (Ser166)	1:2000	CST	3521
phospho-PRAS40 (Thr246)	1:2000	CST	2297
phospho-4E-BP1 (Thr37/46)	1:2000	CST	2855
phospho-rpS6 (Ser235/236)	1:2000	CST	4856
phospho-rpS6 (Ser240/244)	1:2000	CST	2215
Total rpS6	1:2000	CST	2217
phospho-AKT substrate (PAS)	1:2000	CST	9611
Tubulin	1:10000	Sigma	T5168

CST: Cell Signalling Technology

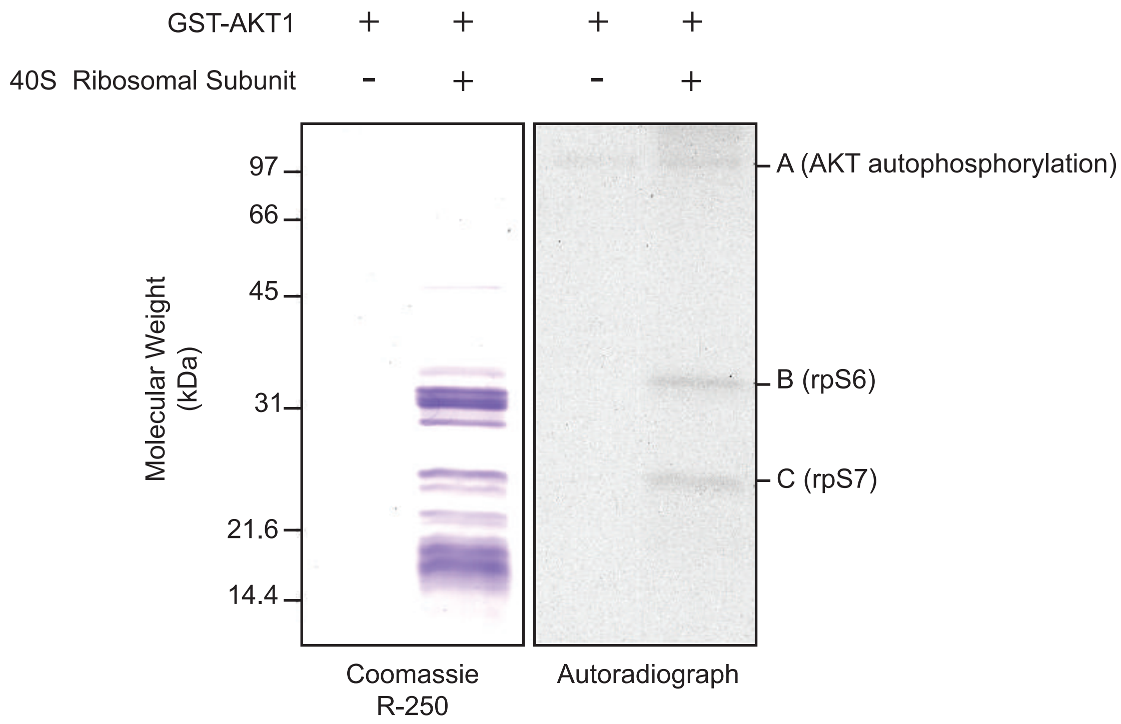

Supplementary Figure 1. rpS6 and rpS7 are in vitro substrates of AKT. Purified GST-AKT1 was incubated with [γ-^{32}P]ATP in the presence and absence of 40S ribosomal subunit isolated from rat liver for 20 min at 30°C. Proteins were resolved by SDS-PAGE and stained with Coomassie R-250. The gel was air dried between cellophane and exposed to hyperfilm for 24hrs. Coomassie R-250 stained proteins corresponding to phosphorylation bands B and C on the autoradiograph were excised and subjected to in gel trypsin digestion before identification by mass spectrometry. Band B was identified to be rpS6, with a mascot score of 281 and a sequence coverage of 24%. Band C was identified to be rpS7, with a mascot score of 120 and sequence coverage of 24%. This figure is a representative of one experiment performed in duplicate.

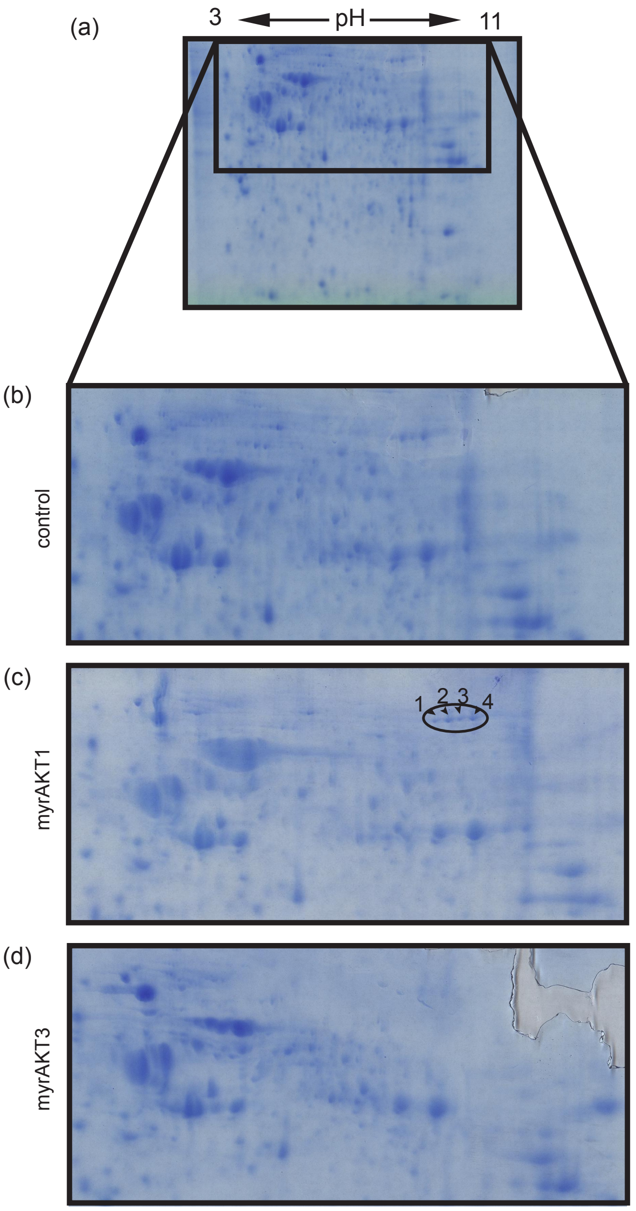

Supplementary Figure 2. Corresponding Coomassie stained 2D gels from Figure 5. HEK293 cells transfected with the pCDNA3 vector as control or expressing similar levels of myrAKT1 or myrAKT3 were serum-starved for 24 hrs prior to harvesting into RLB. Cy2 labelled protein samples (250 µg) were loaded onto 18 cm broad range IPG strips with a non-linear pH range of 3-11, focused and resolved by SDS-PAGE. After 2DGE, gels were stained with Coomassie blue. (a) control. (b – d) enlarged region of 2D gels containing the control, myrAKT1 or myrAKT3 samples respectivley. All spots that were subjected to mass spectrometry analysis were excised from the myrAKT1 gel. The four protein spots (spots 1-4), circled in white were identified as eEF2 by mass spectrometry analysis. n=1

Eukaryotic translation elongation factor 2 (eEF2)

```
  1 MVNFTVDQIR AIMDKKANIR NMSVIAHVDH GKSTLTDSLV CKAGIIASAR
 51 AGETRFTDTR KDEQERCITI KSTAISLFYE LSENDLNFIK QSKDGAGFLI
101 NLIDSPGHVD FSSEVTAALR VTDGALVVVD CVSGVCVQTE TVLRQAIAER
151 IKPVLMMNKM DRALLELQLE PEELYQTFQR IVENVNVIIS TYGEGESGPM
201 GNIMIDPVLG TVGFGSGLHG WAFTLKQFAE MYVAKFAAKG EGQLGPAERA
251 KKVEDMMKKL WGDRYFDPAN GKFSKSATSP EGKKLPRTFC QLILDPIFKV
301 FDAIMNFKKE ETAKLIEKLD IKLDSEDKDK EGKPLLKAVM RRWLPAGDAL
351 LQMITIHLPS PVTAQKYRCE LLYEGPPDDE AAMGIKSCDP KGPLMMYISK
401 MVPTSDKGRF YAFGRVFSGL VSTGLKVRIM GPNYTPGKKE DLYLKPIQRT
451 ILMMGRYVEP IEDVPCGNIV GLVGVDQFLV KTGTITTFEH AHNMRVMKFS
501 VSPVVRVAVE AKNPADLPKL VEGLKRLAKS DPMVQCIIEE SGEHIIAGAG
551 ELHLEICLKD LEEDHACIPI KKSDPVVSYR ETVSEESNVL CLSKSPNKHN
601 RLYMKARPFP DGLAEDIDKG EVSARQELKQ RARYLAEKYE WDVAEARKIW
651 CFGPDGTGPN ILTDITKGVQ YLNEIKDSVV AGFQWATKEG ALCEENMRGV
701 RFDVHDVTLH ADAIHRGGGQ IIPTARRCLY ASVLTAQPRL MEPIYLVEIQ
751 CPEQVVGGIY GVLNRKRGHV FEESQVAGTP MFVVKAYLPV NESFGFTADL
801 RSNTGGQAFP QCVFDHWQIL PGDPFDNSSR PSQVVAETRK RKGLKEGIPA
851 LDNFLDKL
```

Supplementary Figure 3. Identification and sequence analysis of eEF2. Peptides used to identify the proteins (sequence coverage) by mass spectrometry are denoted in red. Partial (RXXS/T) AKT motifs are underlined in green with potential phosphorylated residues indicated by *. Representative sequence analysis of the 4 spots identified as eEF2 from Figure 5 and Supplementary Figure 2. A mascot score of > 181 and sequence coverage of 6-17% were obtained for all 4 protein spots.
